# Challenges in the Development of e-Quit worRx: An iPad App for Smoking Cessation Counseling and Shared Decision Making in Primary Care

**DOI:** 10.2196/11300

**Published:** 2019-03-29

**Authors:** Charles R Doarn, Mary Beth Vonder Meulen, Harini Pallerla, Shauna P Acquavita, Saundra Regan, Nancy Elder, Matthew R Tubb

**Affiliations:** 1 Department of Family and Community Medicine University of Cincinnati Cincinnati, OH United States; 2 School of Social Work, College of Allied Health Sciences University of Cincinnati Cincinnati, OH United States

**Keywords:** mobile health, smoking cessation, primary care, decision aid

## Abstract

**Background:**

Smoking is the leading preventable cause of morbidity and mortality in the United States, killing more than 450,000 Americans. Primary care physicians (PCPs) have a unique opportunity to discuss smoking cessation evidence in a way that enhances patient-initiated change and quit attempts. Patients today are better equipped with technology such as mobile devices than ever before.

**Objective:**

The aim of this study was to evaluate the challenges in developing a tablet-based, evidence-based smoking cessation app to optimize interaction for shared decision making between PCPs and their patients who smoke.

**Methods:**

A group of interprofessional experts developed content and a graphical user interface for the decision aid and reviewed these with several focus groups to determine acceptability and usability in a small population.

**Results:**

Using a storyboard methodology and subject matter experts, a mobile app, e-Quit worRx, was developed through an iterative process. This iterative process helped finalize the content and ergonomics of the app and provided valuable feedback from both patients and provider teams. Once the app was made available, other technical and programmatic challenges arose.

**Conclusions:**

Subject matter experts, although generally amenable to one another’s disciplines, are often challenged with effective interactions, including language, scope, clinical understanding, technology awareness, and expectations. The successful development of this app and its evaluation in a clinical setting highlighted those challenges and reinforced the need for effective communications and team building.

## Introduction

### Background

Smoking is the leading preventable cause of morbidity and mortality in the United States [1]. Each year, smoking kills nearly 450,000 people in the United States and costs almost US $100 billion in health care costs and productivity losses. An estimated 19% of adults in the United States smoke [1]. Although numerous interventions improve the likelihood of successful smoking cessation and the resulting health benefits [2], most smokers relapse or require several interventions and attempts before staying smoke-free [3]. Primary care physicians (PCPs) have an opportunity to discuss smoking cessation evidence in a way that enhances patient-initiated change [4] and quit attempts [5] using new approaches. Unfortunately, although current guidelines summarize the comparative effectiveness of available smoking cessation medications, counseling techniques, and other methods, including smoking cessation apps and social media tools [6-8], physicians discuss cessation with smokers infrequently and underutilize tobacco cessation medications [9,10]. A shared decision-making (SDM) tool can add value to the interchange between a PCP and patient.

Methods that allow physicians to conduct more frequent, efficient tobacco counseling are necessary to disseminate smoking cessation evidence [11-14] and could have a substantial impact, as even brief counseling by a PCP can increase the likelihood of smoking cessation [4]. Decision aids are a method that can assist clinicians and patients in finding motivating, personally effective quit strategies that can be integrated into physician offices where patient-provider discussions about smoking cessation typically occur [15].

### Innovative Approaches

The use of a hand-held electronic tool can increase physicians’ comfort with cessation counseling [16]. Furthermore, SDM has the potential to engage and inform patients and improve quality of care, especially when combined with decision aids and health information technology tools [17]. Integrating such tools can also enable better and timely interaction between a PCP and patient. Tudor-Sfetea et al evaluated mobile health (mHealth) apps in the context of smoking cessation [18]. As a result of using Quit Genius or Smokefree, which are 2 smoking cessation mobile apps in the United Kingdom, this study demonstrated positive preliminary changes in smoking behavior and it was demonstrated that these apps are feasible and potentially effective tools [18].

With so many options for cessation support, it is important for clinicians to personalize evidence-based interventions that are both useful and appealing to patients. During primary care office visits with competing priorities [19], applying patient-centered outcomes research (PCOR) for any given problem can be challenging but can also benefit workflow in the clinic setting by increasing efficiency.

To address these opportunities and challenges, we developed an iPad/tablet-based mHealth decision aid app (e-Quit worRx, University of Cincinnati) to assist PCPs in disseminating PCOR evidence about smoking cessation options and engage in SDM.

The primary objective of this research effort was to develop an acceptable and easy-to-use smoking cessation decision aid that incorporated PCOR evidence into an mHealth tablet-based app called e-Quit worRx. We have discussed the challenges of developing the app through an iterative process.

## Methods

### Project Team

An interdisciplinary team of subject matter experts was formed to complete this project. These experts included specialists in primary care, smoking cessation and social work, health information technology and mHealth, app development and computer programming, and qualitative and primary care practice–based research. Each brought a unique perspective to the overall design of the app both in functionality and utility.

### Conceptual Framework

**Figure 1:**
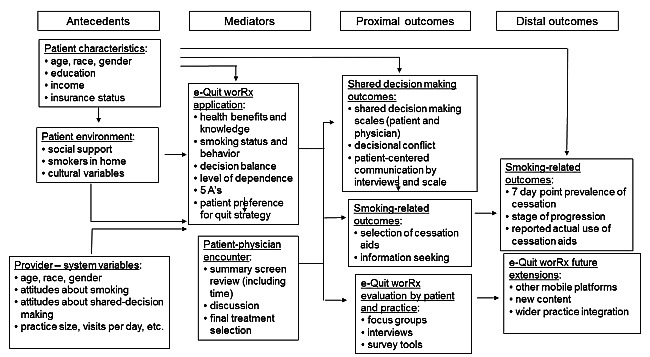
Conceptual framework for shared decision making using an app for evidence-based smoking cessation.

This project was guided by a conceptual framework grounded in SDM and behavioral theories of smoking cessation (eg, stages of change and the 5As—Ask, Advise, Assess, Assist, and Arrange; Figure 1) [20]. This framework includes key determinants, tools, and outcomes that lead to SDM and smoking cessation. We adapted frameworks developed for primary care for smoking cessation counseling [21] and for SDM such as those used in colorectal cancer screening [22] to create a conceptual framework to guide the study innovations, interventions, and outcomes. We aimed to combine theory-driven aspects of smoking cessation (eg, stages of change and self-efficacy) with iPad-based interactive, tailored delivery of PCOR evidence to smokers at the point-of-care (their PCP’s office). The overall goal of the decision aid was to provide evidence-based and patient-centered smoking risk and cessation information to patients. Once developed and acceptable to patients and physicians, the decision aid would then be introduced into a routine office visit while minimizing physician and office staff training and ongoing time commitment.

### Project Design

**Figure 2:**
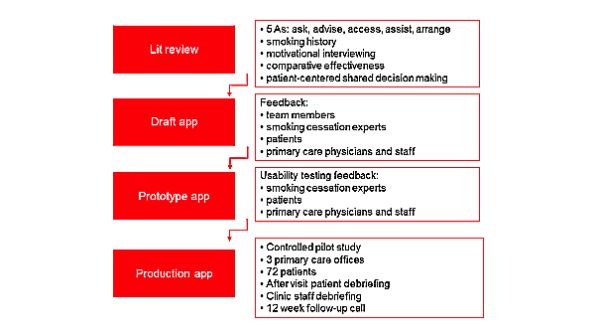
Study flow and app design process.

The project was completed in 3 phases using a design process depicted in Figure 2: (1) development of a storyboard of app content and flow and initial app version; (2) evaluation of the app at various development stages with physicians, medical staff, and patients through an iterative process and app refinement; and (3) clinical pilot testing of the app with patients in the PCPs office. This process was used in the first 2 phases. The third phase will be reported in a separate study.

### Phase 1: Content Development, Initial Feedback, and Storyboarding

Development of a new task or device is often conceptualized through the use of storyboarding [23]. Some processes of app development that are Web based have recently been patented [24]. Iqbal et al have laid out some of the requirements for engineering practices of mobile app development [25]. This process, although challenging, provides an excellent tool for development teams to understand what the final process or device should look like and how it works. The entire research team laid out a storyboard for how the app should flow from a physician and patient perspective.

One of the steps in the storyboarding process is to provide input on how the user will interact with the app. Human factors and ergonomics play a role as well. To accomplish this, separate focus groups with patients and smoking cessation experts were held as well as individual interviews with PCPs and medical support staff. The goal of these initial sessions was to understand what stakeholders wanted and needed to be included in a clinical encounter for smoking cessation. These interviews addressed, as appropriate, previous smoking attempts, previous and desired communication about smoking, use and comfort with electronic media, and knowledge and comfort with evidence-based smoking cessation tools.

Clinical evidence-based content for app development was obtained in a large part from the Smoking Cessation Guidelines for Clinicians [6] and Cochrane reviews [2]. In addition, content from the following were also obtained: the Centers for Disease Control and Prevention’s smokefree.gov website [26] and incorporation of feedback from focus group interviews as well as knowledge from the scientific literature in the following: (1) PCOR studies in the areas of primary care [4,27-29], (2) smoking cessation medications [30], (3) mHealth tools [31-34], and (4) decision aids [35].

#### e-Quit worRx Coding and Design

The team used an iPad platform (iPad 2, Apple), using iOS 7, as the user interface for the decision aid. Code for the app was written in Apple’s Xcode software on a MacMini using the Swift programming language (Apple). In total, 2 graduate students from the university’s computer science program worked with the team to write the code. Prototype app versions were tested on 3 iPad 2 devices.

### Phase 2: Iterative Usability Testing With Stakeholders and End Users

Once a prototype app (version 1.0) was complete, a second round of key informant interviews was completed with patients, clinicians, and clinical support staff. These interviews focused on usability and included a modified System Usability Scale (SUS) as well as a semistructured questionnaire [36]. Interviews touched upon participants’ experiences using the app, recommendations for modifications, and evaluations of specific app components. Initial rounds of testing used concurrent think-aloud techniques to elicit real-time feedback and emotional responses. Later rounds of testing used retrospective think-aloud techniques to assess important metrics, such as accuracy and time, needed to complete tasks on the app.

#### Participants

This study was approved by the Institutional Review Board of the University of Cincinnati.

For the first 2 phases described in this study, our team sought feedback from and recruited key stakeholders including patients and PCPs and primary care office staff (nurses, medical assistants, and office managers) ranging in their comfort and familiarity with technology. Table 1 summarizes the demographics and role of all participants. We also sought feedback from an interdisciplinary team of faculty and staff from the University of Cincinnati with expertise in addressing tobacco cessation.

**Table 1:** Basic demographics and role in Phase 1 and Phase 2. The data reported here reflects those individuals who participated in year 1 in the following: (1) interviews (clinical personnel), (2) focus groups (experts and test patients), and (3) the testing with both clinical personnel and test patients.

Stage, position		Demographics		
		Gender	Age (years)	Race
**Interviews^a^**				
	PCP^b^	F	38	Asian
	PCP	F	31	White
	PCP	F	31	White
	PCP	F	54	White
	PCP	F	49	Asian
	PCP	F	62	White
	Staff registered nurse	F	57	African American
	Staff registered nurse	F	38	White
	Staff MA^c^	F	43	White
	Staff MA	F	36	African American
	Staff MA	F	33	White
**Expert focus group**				
	Assistant professor	F	42	White
	PhD researcher	M	42	White
	Professor, clinical pharmacist specialist	F	58	White
	Nicotine expert	M	(Missing)	African American
	Professor	F	47	White
	Director, addiction division, Veterans Affairs	M	49	White
	Tobacco treatment specialist (retired)	F	71	White
	Coordinator/lung cancer screening	F	58	White
	Case manager/lung cancer screening	F	43	White
**Patient focus group (smoker)**				
	Yes	F	69	White
	Yes	M	66	White
	Yes	M	39	White
	Yes	F	29	White
	Yes	F	33	White
**App testing with clinical personnel**				
	PCP	F	30	African American
	PCP	F	54	White
	PCP	M	40	White
	Staff registered nurse	F	57	White
	Staff registered nurse	F	38	White
**App testing with patients**				
	—^d^	M	30	White
	—	F	52	White
	—	M	66	White
	—	F	59	White
	—	M	29	White
	—	M	47	White
	—	M	55	White

^a^Interviews are from clinical personnel from 3 clinical sites.

^b^PCP: primary care physician.

^c^MA: medical assistant.

^d^—: not applicable.

Patient participants were recruited from the target practices for the eventual pilot trial and recruitment guidelines were in line with previous similar studies [27]. Nonpatient participants were recruited using the snowball technique, beginning with physicians and experts known to the research team, who were then asked to recommend others who could speak on the topic of interest and so on.

A mixed-methods approach was incorporated for app design. The primary outcome was to determine usability. Data sources included qualitative feedback from semistructured interviews and focus groups with key stakeholders, SUS results, and feedback and discussions among our research team members. Interviews were conducted until saturation was achieved—no new ideas were being brought forward [37]. The stakeholders included 10 patients, 7 clinical support staff members (medical assistants and nurses), 8 primary care providers (physicians and advanced practice nurses), and 9 smoking cessation experts.

## Results

### Overview

**Figure 3:**
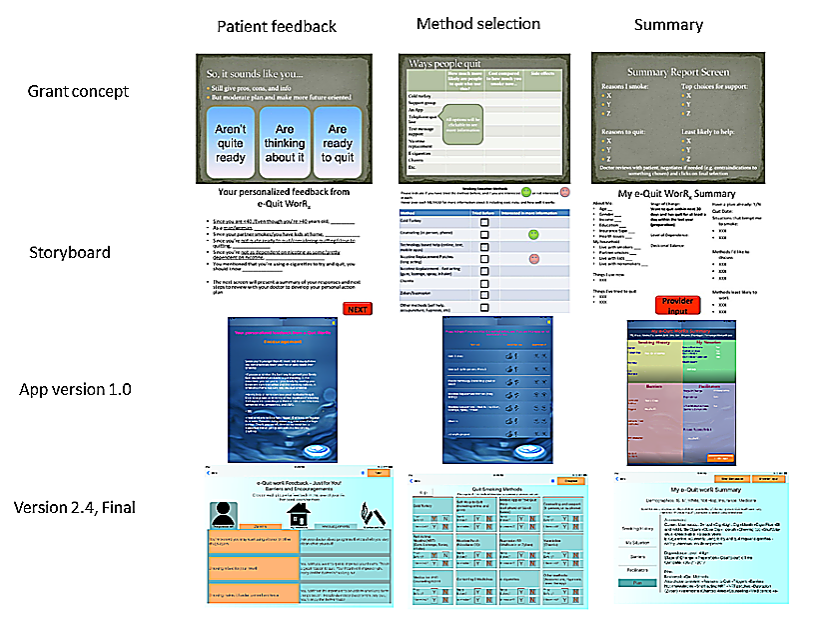
Development of select e-Quit worRx screenshots from Grant Concept to Storyboard, through iterative app versions.

Stakeholder feedback was obtained iteratively before the first app version and with each of the 5 app versions (Table 2). During each increment, changes were made in app content, appearance, and flow based on detailed feedback from the focus groups with changes between versions ranging from relatively minor content revisions or additions to major changes to the graphical user interface. Figure 3 illustrates representative screenshots showing how the app content and appearance changed from version to version.

### Readability

**Figure 4:**
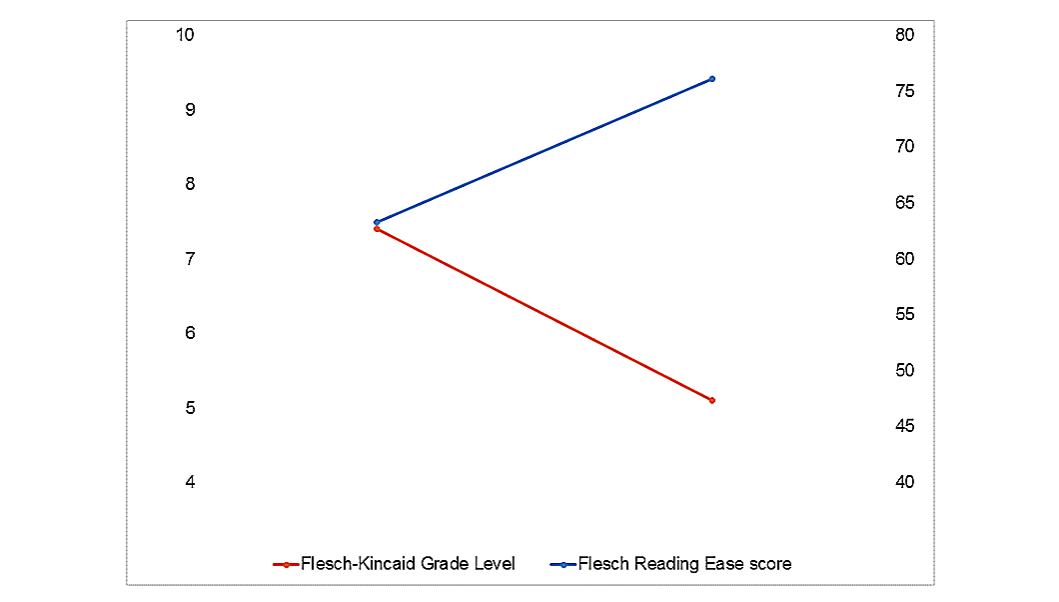
Grade level (left scale) and Reading Ease score (right scale) for app version 2.2 (left) and 2.3 (right).

Testing of version 2.2 produced feedback that the literacy level was too high for the clinical populations served. A literacy evaluation revealed that the initial text averaged a seventh-grade reading level. Between app versions 2.2 and 2.3, text edits were made screen-by-screen and each focused on improving readability to a fifth-grade reading level (Figure 4 illustrates the decrease in the reading level and an increase in ease of reading; enabling a wider audience to understand the wording and phraseology of the app).

### Usability

Usability, as assessed with the SUS, increased with each version for a final of 90/100, above 65 was considered usable (Table 3). After iterative usability testing, a final app version was ready for pilot testing in the clinical setting.

### Description of e-Quit worRx

The app-based decision aid *e-Quit worRx* has several key components, including collecting (1) a comprehensive smoking history, (2) personal reasons for and against smoking, (3) barriers and facilitators to quitting, (4) describing treatment options, including their level of evidence, risks, and costs, and finally (5) summarizing content to aid in SDM. The graphical user interface was unidirectional but used branching logic based on user input. The app begins with a splash screen followed by a secure login screen so that user data were encrypted on the device. Before each participant used the device, the research assistant or principal investigator logged in for the user. The system then randomly assigned a number for each participant.

The app was designed to personalize users’ examination of the positive and negative effects of smoking and increase their knowledge of smoking cessation treatment options.

Treatment options included first-line medications, therapy including local cognitive behavioral therapy providers, and other treatments such as telephone quit lines and mHealth tools.

A summary screen was saved, entirely customized to an individual’s input, to facilitate discussion with their PCP. The summary screen included personalized information derived from their responses. In addition to summarizing their personal considerations about the pros and cons of smoking, it summarized interest in the various cessation aids. The app included a provider input screen, where a plan was selected and an exit interview was to be completed by the research team after the clinical encounter.

The app collects basic demographics, including race, sex, income, age, frequency of smoking, and desire to quit for control groups and intervention groups.

**Table 2:** Themes from qualitative analysis of focus groups and usability testing.

Stage, position, theme			Representative quote
**App development**			
	**Patients**		
		Present treatment options in the app	“Present different treatment options, main risks of things, like with Chantix it is a plus and minus because it is actually very good at helping some people but it can have some nasty side effects.”
		Present cost in the app	“There was a cigarette calculator thing that I went online and you put in how many years you have smoked and how much and with that you could have bought a luxury car with all that money. Something along those lines.”
	**Physicians**		
		Gauging their readiness	“I do not necessarily go through the formal stage criteria but after 20 years you have some idea of what phase someone is in. That helps to see if they are ready to quit-something like that.”
		Time to complete the app information	“If you are delaying my visit because they are out there filling this thing out and we are calling them and they are not done with their survey, then it would be a problem. Has to be done in waiting room or exam room before I get there.”
	**Registered nurses and medical assistants**		
		What has worked in the past and what has not would be helpful	“I say the doctor has lots of materials and I ask them what they have been trying to do, what worked and what did not, it would be helpful to know that about the patient.”
**App Testing**			
	**Patients**		
		(V^a^1.0) Visual and ease-of-use	“More uniform text style, better contrast, too dark of a background, visually challenging-just kind of drives me crazy, lots of mental gymnastics that make you leap back and forth.”
		(V2.1) Customized feedback and knowledge	“I could tell that the feedback was customized at the end, kind of surprised, and I liked increased knowledge about cost of smoking and personal barriers to quitting.”
	**Physician**		
		(V2.3.1) Evidence based methods	“Evidence-based methods are helpful, pros and cons, cost is helpful.”
		(V2.3.1) Saves time for visits about smoking or patient wants to discuss smoking	“Cuts back on me asking all the questions, gives you some tools that might be helpful, and app is a conversation starter.”
	**Medical assistant**		
		(V2.3.1) Time for filling out app	“It was not disruptive, It went well and we still stayed on schedule.”

^a^V: version.

User input, including audio capture from the exit interview, is temporarily stored on the device in an app-based database until the session is complete. Data are then uploaded wirelessly to a Health Insurance Portability and Accountability Act (HIPAA)–compliant Research Electronic Data Capture (REDCap) database.

Overcoming challenges faced in the design process, a user friendly and acceptable iPad app-based decision aid for use in primary care offices was created. Challenges enumerated in Textbox 1 include navigating requests to our coders for repeated changes to both content and design, resolving conflicting feedback from our diverse group of stakeholders and even within our study group, realizing the time intensity of editing content and code, and integration into a clinical setting. We observed challenges between engineers and physicians that required management and interaction to remain on target.

### Limitations

Our study had a few predicted and unforeseen limitations. Although we were able to upload content into the REDCap database, we were not, as we had foreseen, able to fully integrate the app into the electronic health record (EHR) so that patient selections and chosen interventions would populate into the medical record. Our Health Information Technology Department reported that both the timeframe and budget were far too small for this.

Another limitation was that we were unable to create a generic app framework so that clinical content could be swapped out to create decision aid apps for other clinical scenarios, for example, diabetes medicine selection. This was an initial goal, but during the app design process, we made a decision to choose personalization for the patient over future generalizability.

We also discovered that not only would much more work have to go into making our iPad app compatible with iPhones or even Android devices, but we had to choose landscape or portrait display on the iPad instead of allowing the user to decide to ensure the app displayed correctly on the screen. Making the display orientation neutral would have required more programming time than allowed for our study.

**Table 3:** System Usability Scale across app versions.

App version	Usability
1.0	77.5
2.1	77.5
2.2	80.8
2.3	82.5
2.4	90.0

**Examples of challenges by area.**

- Content development:
  1. Deciding what evidence to include when literature is conflicting
  2. Time consuming edits of text screen by screen that required medical knowledge
  3. Fidelity of detail on smoking cessation—physician versus patient confusion to the end user
- Feedback:
  1. The process was iterative and feedback was influenced by level of understanding
  2. Technical prowess
  3. Visual appearance and appeal of the app
  4. Too many colors and busyness of screen make is chaotic
  5. Compromising when feedback from different sources conflicted
- Coding and working with programming engineers:
  1. Challenging dialogue between medical and engineering/computer programming personnel
  2. Acceptability of multiple iterative change requests versus desire of programmers to get a full request, complete it once and be done
  3. Language barrier—style *British English* rather than *American English*
- Health Information Technology and clinical integration:
  1. In ability to fully integrate with electronic health record (Epic) at clinical sites to update patient’s record
  2. Network access at clinical sites—workaround devised
  3. Printer access—had to purchase new printers

We were able to integrate into the clinic sites in several ways. We gained access to the network and internet connection, allowing real-time secure data transfer to our database. We enabled automated HIPAA-compliant email messaging to patients at the end of the session summarizing the interventions chosen, and we built new matching templates (ie, SmartPhrases and a SmartSet order set) for our EHR so that providers could quickly copy over patient selections from the study. Finally, although the existing clinic printers could not be used to print from our app, we placed AirPrint-enabled printers at each site to allow printing summaries for patients and PCPs.

## Discussion

mHealth is no longer a novel approach or tool for health care. Tools have been developed and tested for smoking cessation [15,27-30] and other clinical conditions. Today, patients are more engaged in the management of their health than ever before. SDM tools are important in the management of disease, and some health care is actually moving toward the patient-centered home [37-39]. Acceptance of computer-based tools in addressing patients’ needs whether in the home or exam room are more acceptable today as well [40-43].

As demonstrated by this study, development of mHealth solutions is time-consuming and challenging. The life cycle of such devices is short-lived and must be upgradeable with changes in software versions, operating systems, and consumer needs. Nevertheless, health care will continue to integrate technologies such as e-Quit worRx into the management of a patient’s health.

This research effort was focused on the development of an app for smoking cessation SDM using an iPad-based platform. A fully functional system was developed over several iterations. There were several challenges in the development phase. Insufficient funding limited the level of computer programming expertise. Although the students were adept at programming, the complexity of the software used to develop the app, concomitant with automatic Apple Operating System upgrades and the varying levels of communications, provided a significant challenge. Of the observations noted, 1 was the dichotomy in conversation between the graduate student programmers and clinicians. Multiple discussions and interaction with all of the research team provided resolution and a functional app, which was able to be used in clinical testing in year 2.

## Abbreviations

EHR: electronic health record
HIPAA: Health Insurance Portability and Accountability Act
mHealth: mobile health
PCOR: patient-centered outcomes research
PCP: primary care physician
REDCap: Research Electronic Data Capture
SDM: shared decision making
SUS: System Usability Scale

## References

[1] Centers for Disease Control and Prevention.

[2] Hartmann-Boyce J, Stead L, Cahill K, Lancaster T (2014). Efficacy of interventions to combat tobacco addiction: Cochrane update of 2013 reviews. Addiction.

[3] Brandon TH, Tiffany ST, Baker TB (1986). The process of smoking relapse. NIDA Res Monogr.

[4] Gorin SS, Heck JE (2004). Meta-analysis of the efficacy of tobacco counseling by health care providers. Cancer Epidemiol Biomarkers Prev.

[5] Zwar NA, Richmond RL (2006). Role of the general practitioner in smoking cessation. Drug Alcohol Rev.

[6] (2008). Agency for Healthcare Research and Quality.

[7] Baskerville NB, Struik LL, Guindon GE, Norman CD, Whittaker R, Burns C, Hammond D, Dash D, Brown KS (2018). Effect of a Mobile Phone Intervention on Quitting Smoking in a Young Adult Population of Smokers: Randomized Controlled Trial. JMIR Mhealth Uhealth.

[8] McKelvey K, Ramo D (2018). Conversation Within a Facebook Smoking Cessation Intervention Trial For Young Adults (Tobacco Status Project): Qualitative Analysis. JMIR Form Res.

[9] Thorndike AN, Regan S, Rigotti NA (2007). The treatment of smoking by US physicians during ambulatory visits: 1994 2003. Am J Public Health.

[10] Goldstein MG, Niaura R, Willey-Lessne C, DePue J, Eaton C, Rakowski W, Dubé C (1997). Physicians counseling smokers. A population-based survey of patients' perceptions of health care provider-delivered smoking cessation interventions. Arch Intern Med.

[11] Thorndike AN, Rigotti NA, Stafford RS, Singer DE (1998). National patterns in the treatment of smokers by physicians. J Am Med Assoc.

[12] Doescher MP, Saver BG (2000). Physicians' advice to quit smoking. The glass remains half empty. J Fam Pract.

[13] Ellerbeck EF, Ahluwalia JS, Jolicoeur DG, Gladden J, Mosier MC (2001). Direct observation of smoking cessation activities in primary care practice. J Fam Pract.

[14] Gilpin EA, Pierce JP, Johnson M, Bal D (1993). Physician advice to quit smoking: results from the 1990 California Tobacco Survey. J Gen Intern Med.

[15] Agarwal SD, Kerwin M, Meindertsma J, Wolf AM (2018). A novel decision aid to encourage smoking cessation among patients at an urban safety net clinic. Prev Chronic Dis.

[16] Strayer SM, Heim SW, Rollins LK, Bovbjerg ML, Nadkarni M, Waters DB, Hauck FR, Schorling JB (2013). Improving smoking cessation counseling using a point-of-care health intervention tool (IT): from the Virginia Practice Support and Research Network (VaPSRN). J Am Board Fam Med.

[17] Fowler FJ, Levin CA, Sepucha KR (2011). Informing and involving patients to improve the quality of medical decisions. Health Aff (Millwood).

[18] Tudor-Sfetea C, Rabee R, Najim M, Amin N, Chadha M, Jain M, Karia K, Kothari V, Patel T, Suseeharan M, Ahmed M, Sherwani Y, Siddiqui S, Lin Y, Eisingerich AB (2018). Evaluation of two mobile health apps in the context of smoking cessation: qualitative study of cognitive behavioral therapy (CBT) versus non-CBT-based digital solutions. JMIR Mhealth Uhealth.

[19] Jaén CR, Stange KC, Nutting PA (1994). Competing demands of primary care: a model for the delivery of clinical preventive services. J Fam Pract.

[20] Vidrine JI, Shete S, Cao Y, Greisinger A, Harmonson P, Sharp B, Miles L, Zbikowski SM, Wetter DW (2013). Ask-Advise-Connect: a new approach to smoking treatment delivery in health care settings. JAMA Intern Med.

[21] Strayer SM, Martindale JR, Pelletier SL, Rais S, Powell J, Schorling JB (2011). Development and evaluation of an instrument for assessing brief behavioral change interventions. Patient Educ Couns.

[22] Christy SM, Rawl SM (2013). Shared decision-making about colorectal cancer screening: a conceptual framework to guide research. Patient Educ Couns.

[23] Nosseir A, Flood D, Harrison R, Ibrahim O IEEE Xplore Digital Library.

[24] Brisebois M, Drummond B, Mehta A, Chene M, Flannigan M (2017). Google Patents.

[25] Iqbal J, Ahmad R, Nasir M, Khan M (2017). Significant requirements engineering practices for outsourced mobile application development. J Inform Sci Eng.

[26] (2018). Smokefree.gov.

[27] Unrod M, Smith M, Spring B, DePue J, Redd W, Winkel G (2007). Randomized controlled trial of a computer- based, tailored intervention to increase smoking cessation counseling by primary care physicians. J Gen Intern Med.

[28] Linder JA, Rigotti NA, Schneider LI, Kelley JH, Brawarsky P, Haas JS (2009). An electronic health record-based intervention to improve tobacco treatment in primary care: a cluster-randomized controlled trial. Arch Intern Med.

[29] Goldberg D, Hoffman A, Anel D (2002). Understanding people who smoke and how they change: a foundation for smoking cessation in primary care, part 1. Dis Mon.

[30] Cornuz J, Gilbert A, Pinget C, McDonald P, Slama K, Salto E, Paccaud F (2006). Cost-effectiveness of pharmacotherapies for nicotine dependence in primary care settings: a multinational comparison. Tob Control.

[31] Marcy T, Skelly J, Shiffman R, Flynn B (2005). Facilitating adherence to the tobacco use treatment guideline with computer-mediated decision support systems: physician and clinic office manager perspectives. Prev Med.

[32] Chen Y, Madan J, Welton N, Yahaya I, Aveyard P, Bauld L, Wang D, Fry-Smith A, Munafò MR (2012). Effectiveness and cost-effectiveness of computer and other electronic aids for smoking cessation: a systematic review and network meta-analysis. Health Technol Assess.

[33] Michel G, Marcy T, Shiffman R (2005). A wireless, handheld decision support system to promote smoking cessation in primary care. AMIA Annu Symp Proc.

[34] Marcy TW, Kaplan B, Connolly SW, Michel G, Shiffman RN, Flynn BS (2008). Developing a decision support system for tobacco use counselling using primary care physicians. Inform Prim Care.

[35] Bakken S, Roberts W, Chen E, Dilone J, Lee N, Mendonca E, Markatou M (2007). PDA-based informatics strategies for tobacco use screening and smoking cessation management: a case study. Stud Health Technol Inform.

[36] Ithnin M, Mohd Rani Mohd Dzulkhairi, Abd Latif Zuraidah, Kani P, Syaiful A, Nor Aripin Khairun Nain, Tengku Mohd Tengku Amatullah Madeehah (2017). Mobile App Design, Development, and Publication for Adverse Drug Reaction Assessments of Causality, Severity, and Preventability. JMIR Mhealth Uhealth.

[37] Sauro J (2011). MeasuringU.

[38] Willemsen M, Wiebing M, van Emst A, Zeeman G (2006). Helping smokers to decide on the use of efficacious smoking cessation methods: a randomized controlled trial of a decision aid. Addiction.

[39] Nutting P, Crabtree B, Miller W, Stange K, Stewart E, Jaen C (2011). Transforming physician practices to patient-centered medical homes: lessons from the national demonstration project. Health Aff (Millwood).

[40] Barry MJ, Edgman-Levitan S (2012). Shared decision making--pinnacle of patient-centered care. N Engl J Med.

[41] Oshima LE, Emanuel EJ (2013). Shared decision making to improve care and reduce costs. N Engl J Med.

[42] Strayer SM, Semler MW, Kington ML, Tanabe KO (2010). Patient attitudes toward physician use of tablet computers in the exam room. Fam Med.

[43] Sciamanna C, Marcus B, Goldstein M, Lawrence K, Schwartz S, Bock B, Graham A, Ahern D (2004). Feasibility of incorporating computer-tailored health behaviour communications in primary care settings. Inform Prim Care.

